# *Urtica pilulifera L.* seed extract promotes folliculogenesis and alleviates the diminished ovarian reserve in the Balb/c mice model: An experimental study

**DOI:** 10.18502/ijrm.v22i2.15708

**Published:** 2024-03-25

**Authors:** Sharareh Hekmat, Mohammad Sharifzadeh, Tayebeh Toliyat, Roghayeh Savary Kouzehkonan, Mozhgan Mehri Ardestani, Malihe Tabarrai, Seyede Nargess Sadati Lamardi

**Affiliations:** ^1^Department of Traditional Pharmacy, School of Traditional Persian Medicine, Tehran University of Medical Sciences, Tehran, Iran.; ^2^Department of Pharmacology and Toxicology, Faculty of Pharmacy, Tehran University of Medical Sciences, Tehran, Iran.; ^3^Department of Pharmaceutics, Faculty of Pharmacy, Tehran University of Medical Sciences, Tehran, Iran.; ^4^Department of Clinical Pharmacy, Faculty of Pharmacy, Tehran University of Medical Sciences, Tehran, Iran.; ^5^Department of Persian Medicine, Faculty of Medicine, AJA University of Medical Sciences, Tehran, Iran.; ^6^Department of Persian Medicine, School of Traditional Persian Medicine, Tehran University of Medical Sciences, Tehran, Iran.

**Keywords:** Apoptosis, Fatty acids, Female infertility, Herbal medicine, Persian medicine, Oxidative stress.

## Abstract

**Background:**

*Urtica pilulifera L.* seed (UPS) is a Persian traditional medicine prescription that positively affects female infertility.

**Objective:**

This study aimed to evaluate the beneficial effects of UPS on a diminished ovarian reserve (DOR) model induced by cyclophosphamide in Balb/c mice.

**Materials and Methods:**

A single intraperitoneal (75 mg/kg) of cyclophosphamide was administered to establish a DOR model. 25 female Balb/c mice (6–8 wk, 25 ± 2 gr) were randomly divided into 5 groups (n = 5/each), including control (normal saline), model (DOR), DOR+50, DOR+100, and DOR+200 (mg/kg UPS, gavage) groups for 14 days. The levels of follicle-stimulating hormone, luteinizing hormone, estradiol, malondialdehyde, superoxide dismutases, apoptosis, and histopathological alterations were analyzed. Gas chromatography-mass spectrometry analysis was performed to identify the phytochemicals of the UPS.

**Results:**

It was observed that the UPS extract reduced malondialdehyde concentration and apoptosis in the DOR model as well as enhanced superoxide dismutases activity in the ovaries in a dose-dependent manner. Moreover, it exerted a modulatory effect on steroidal hormones such as follicle-stimulating hormone, luteinizing hormone, and estradiol. The histopathological analysis revealed the therapeutic potential of the UPS extract. The main chemical components of UPS were linoleic acid (59.25%), n-hexadecanoic acid (10.36%), and oleic acid (8.29%).

**Conclusion:**

The results indicated that the UPS extract has therapeutic potential in the DOR model. This potential is attributed to the reduction of oxidative stress, modulation of apoptosis, and regulation of steroidal hormones that may be associated with the observed beneficial effects of fatty acids on fertility improvement.

## 1. Introduction 

Infertility is a health problem described as the inability to conceive a baby after a year of unprotected sexual intercourse. It approximately affects 20–30% of the females of reproductive age (1) and the rate of female infertility is about 15% (2). Female infertility can be attributed to various causes, such as issues related to ovulation (3). The ovarian reserve has a prominent role in pregnancy achievement and reflects the follicular pool and quality of oocytes. A decline in the ovarian reserve occurs progressively with age due to increased reactive oxygen species (ROS) production, exposure to toxins, and genetic alterations (4, 5).

Diminished ovarian reserve (DOR) is used to indicate the clinical importance of this reduction. Several risk factors have been proposed for DOR, such as idiopathic causes, smoking, chemotherapy, and radiotherapy. The prevalence of this complication has been increasing in recent years, rising from 19% in 2004 to 26% in 2011 (6, 7). Various treatment strategies have been proposed for addressing DOR, including the use of medicinal supplements such as growth hormones, dehydroepiandrosterone, clomiphene citrate, melatonin, and letrozole (8). However, it is important to note that long-term use of some of these agents may carry potential risks, including an increased risk of diseases such as endometrial and breast cancers (9).

Herbal medicines have gained increased acceptance among consumers due to their perceived lower toxicity and cost when compared to synthetic drugs (10). Therefore, harnessing the potential of traditional medicine can be a promising option for the identification and utilization of medicinal compounds to treat DOR. *Urtica pilulifera* *L.* (*U. pilulifera*) is an annual plant from the Urticaceae family cultivated in different parts of the world. In folk medicine, *U. pilulifera* is believed to treat various complications such as hypertension, hyperglycemia, and inflammation (11). The *U. pilulifera* seed (UPS) extract contains fatty acids such as linoleic and linolenic acids. In addition, it has antioxidant properties due to a high content of phenolic compounds and tocopherols (12).

It is believed in Persian traditional medicine that dystemperament (*sue' mizaj*), along with other factors such as obstructive causes, can potentially hinder female fertility (13). According to various resources in Persian traditional medicine, one of the most common causes of infertility among females is attributed to changes in the temperament(*mizaj*) of the uterus, leading to coldness (*burudat*). In recent studies, the most common temperament found in patients with infertility complaints was cold and wet. It was also confirmed that the temperament of the uterus is related to the hot/cold temperament of the body and pelvic width (14, 15). Folk remedy emphasizes the hot (*harr*) property of *U. pilulifera*, suggesting that it can potentially increase the quantity of semen and enhance sexual libido and arousal in both males and females. Therefore, it can be considered a treatment option for infertility (16).

The present study was conducted to investigate the therapeutic potential of *U. pilulifera *in cyclophosphamide (CTX)-induced DOR model in Balb/c mice.

## 2. Materials and Methods

### Chemicals and reagents 

In this experimental study, cyclophosphamide (CTX) was purchased from Baxter Oncology, Germany. H_2_O_2_, Triton x100, proteinase K, hematoxylin, eosin, and other chemicals were purchased from Sigma-Aldrich (St Louis, MO, USA). The enzyme terminal deoxynucleotide transferase (TdT) was purchased from Roche, USA. The enzyme-linked immunosorbent assay (ELISA) kits for measuring estradiol, follicle-stimulating hormone (FSH), luteinizing hormone (LH), superoxide dismutase (SOD), and malondialdehyde (MDA) were purchased from ZellBio GmbH, Germany.

### Preparation of plant seed and extraction

The UPS intact seeds were obtained from a local, herbal product, market in Tehran, Iran. After authentication, they were stored at the herbarium of the Faculty of Pharmacy, Tehran University of Medicinal Sciences, Tehran, Iran (voucher number: 1740). 3 kg UPS were ground into a powder using a grinder. The powdered seeds were then soaked in 70% ethanol. After every 24 hr, the extract was collected from the soaked UPS powder, and fresh solvent (70% ethanol) was added to continue the extraction process. This cycle of collection and addition of fresh solvent was repeated 3 times. The UPS extract obtained from the soaking process was concentrated using a rotary evaporator at 40 C and subsequently dried in a vacuum oven. The extraction yield achieved was 10%. The resulting product, the total hydroalcoholic extract of UPS, was utilized for both animals and in vitro assays.

### Gas chromatography-mass spectrometry analysis

#### UPS extraction 

The seed powder was subjected to extraction using n-hexane (Merck, Germany) in a Soxhlet apparatus for 4 hr. After extraction, the solvent was concentrated using a rotary evaporator at 40ºC. The extract yield of the seeds was 25% (w/w).

#### GC-MS conditions

The gas chromatography-mass spectrometry (GC-MS)device model 7890 equipped with MS model 5975 (Agilent, USA) split/spitless input, which incorporates a quadrupole-type mass spectrometer, was used for the analysis. The separation process was performed using a polydimethylsiloxane capillary column (HP-5 MS, 5% phenyl-95%) measuring 30 × 0.25 mm ID. The column was made from silica and had a film thickness of 0.25 µm. Perfluoro-tert-butylamine was used for mass spectrometer calibration. Secondary-ion mass spectrometry analysis was used for each target species.

In this mode, the device selectively detects and measures a specific set of m/z values that are determined by the user and have the highest frequency. By focusing on these specific m/z values instead of scanning a wide range, the SIM mode enhances sensitivity and allows for more precise and targeted analysis. For optimal separation, helium with a purity of 99.99% was employed as the carrier gas at a flow rate of 1 ml/min. The device was operated in splitless mode at the inlet, facilitating the best possible separation during the analysis process. The device software was MSD ChemStation version E.02.01.1177 (Agilent Technologies, Santa Clara, CA, USA). The chemical components of the UPS hexane extract were identified by comparing their retention time and mass weight with those of authentic samples obtained by GC, as well as by analyzing the mass spectra using the Wiley and Nist databases through GC-MS.

### Animals and treatment

25 female Balb/c mice (6–8 wk, 25 ± 2 gr) were procured from the Pasteur Institute, Tehran, Iran. All animals were housed in standard cages in the Laboratory Animal Center of the Faculty of Pharmacy, Tehran University of Medical Science, Tehran, Iran. The mice were housed in a controlled temperature (23 ± 3 C), humidity (50 ± 10%), and a 12 hr light and dark cycle. Throughout the study, the animals had unrestricted access to appropriate food and tap water. The cages of the animals were kept separately. The animals were kept in the laboratory a week before the experiment to adapt to the conditions and ensure the estrous cycle. To maintain the estrous cycle in the mice, pieces of straw that were stained with the urine of male mice were transferred to the cages of the studied mice.

25 mice with normal estrous cycle, via vaginal smear checkup, were randomly divided into 5 groups (n = 5/each), including control, model group (DOR), DOR+UPS 50 (DOR and 50 mg/kg UPS), DOR+UPS 100 (DOR and 100 mg/kg UPS), and DOR+UPS 200 (DOR and 200 mg/kg UPS) groups. To establish a DOR model, a single intraperitoneal dose of CTX was administered at a dose of 75 mg/kg. All mice in the DOR, DOR+50, DOR+100, and DOR+200 groups received a single dose of CTX, dissolved in saline, intraperitoneally, whereas in control group an equal volume of saline was injected intraperitoneally. The DOR model was established, after 14 days of CTX administration, according to the estrous cycle of animals that was prolonged to 8–10 days (17).

The animals in UPS groups received 0.2 ml of the seed extract by gavage, while the control and DOR groups received an equal volume of normal saline for 14 days and were then euthanized by carbon dioxide (CO ${\;}_{2}$ ). Blood samples were collected by cardiac puncture. The dosage of UPS used in the study was determined based on the safety and efficacy observed in a pilot study. No complications or deaths were observed during the experiment.

### Body, ovarian, and uterus weight

Before and after UPS treatment, the body weight of each mouse was recorded every week until the time of sacrifice. Upon sacrifice, the ovaries and uterus of the mice were isolated and weighed individually.

### Serum hormone level

The serum hormone levels of animals in all groups were evaluated to gain a better understanding of ovarian reserve. ELISA kits were utilized to measure the serum concentrations of FSH, LH, and estradiol. All procedures were conducted following the manufacturer's instructions.

### SOD and MDA assay 

The SOD activity, as an antioxidant enzyme, and the MDA level were assessed in the serum using ELISA kits according to the manufacturer's instructions.

### Apoptosis assay by terminal deoxynucleotidyl transferase deoxyuridine triphosphate nick end labeling (TUNEL) test

To analyze apoptotic cell death, TUNEL test was used (18, 19). Briefly, after the deparaffinization, the slides were washed with phosphate-buffered saline (PBS). A mixture of H ${\;}_{2}$ O ${\;}_{2}$ and methanol (1:9 ratio) was applied to the samples for 10 min. The slides were washed with PBS for 5 min. Proteinase K was applied to the samples for 30 min at 37 C, followed by the addition of 0.3% triton to enhance nucleus permeability. After washing with PBS, the enzyme terminal deoxynucleotide transferase (TdT) was applied to the samples for 2 hr at 37 C. Finally, the nuclei were stained with DAPI (4',6-Diamidino-2-phenylindole), and apoptotic cells were evaluated using an Olympus fluorescent microscope (20).

### Histological analysis of ovary and uterus

Following the surgical procedure, the ovaries and uterine tissues were promptly fixed in 10% buffered formalin and prepared for paraffin embedding. Subsequently, sections with a diameter of 5 μm were obtained and stained with hematoxylin and eosin (H&E) for microscopic analysis (18). Masson's trichrome is a traditional stain used to detect collagen in histologic sections. In brief, after deparaffinization, the slides were submerged into descending grades of alcohol.

The slides were subjected to autoclaving at 56 C in Bowen's solution for 1 hr. Following that the slides were thoroughly washed with running water until the yellow coloration was eliminated. Subsequently, the slides were immersed in Weigert's hematoxylin for 10–15 min. Afterward, the slides were washed with running water for 10 min and rinsed with distilled water. The slides were then placed in Biebrich scarlet-acid fuchsin solution for 10–15 min. A solution of phosphotungstic and phosphomolybdic acids was applied to the slide, covering it for approximately 10–15 min. Subsequently, the slide was immersed in an aniline blue solution for 20–25 min. The samples were rinsed with distilled water and then immersed in 1% acetic acid solution for approximately 2–5 min. Finally, the slides were washed again with distilled water and subjected to dehydration using alcohol (19). In H&E stained samples, follicles were classified as described previously, the existence of oocytes enclosed by a single layer of granulosa cells is known as a primordial follicle, the observation of oocytes surrounded by 2 or more layers of granulosa cells is considered as a preantral follicle, antral follicle comprises oocytes with liquid between the granulosa cells, Graff follicle includes a follicle with a largely fluid-filled antrum, corpus luteum is created by the rupture of the Graff follicle and a follicle with impaired morphological integrity is known as an atretic follicle (21). To avoid double counting, follicles were counted only when the oocyte nucleus was present. The normal follicles were considered and counted to find the masses and percentages of primordial, primary, and secondary (22). Histomorphometry of the uterus was observed by measuring the diameter of the uterine glandular and muscular layer using a microscope aided by ToupView software. All histological sections were performed in @Histogenotec Basic Medical Science Research Center.

### Ethical considerations

All experimental procedures were approved by the Tehran University of Medical Sciences, Tehran, Iran Ethics Committee (Code: IR.TUMS.MEDICINE.REC.1399.744). This study was in accordance with the National Institute of Health Guidelines for the care and use of laboratory animals (23).

### Statistical analysis

All experiments are expressed as mean ± SD. The Bartlett test was used to determine variance homogeneity and the Kolmogorov-Smirnov test was applied to determine normal distribution of data. Comparison between groups was made using one-way ANOVA, followed by Tukey's post-test. P-values < 0.05 were considered statistically significant. Statistical analyses were performed using the GraphPad Prism 8.

## 3. Results 

### GC-MS analysis of UPS 

The GC-MS analysis data of the UPS oil are shown in table I. The main chemical components of UPS oil were linoleic acid (59.25%), n-hexadecanoic (palmitic) acid (10.36%), and oleic acid (8.29%).

### UPS treatment effects on the body, uterus, and ovarian weight

**Figure 1:**
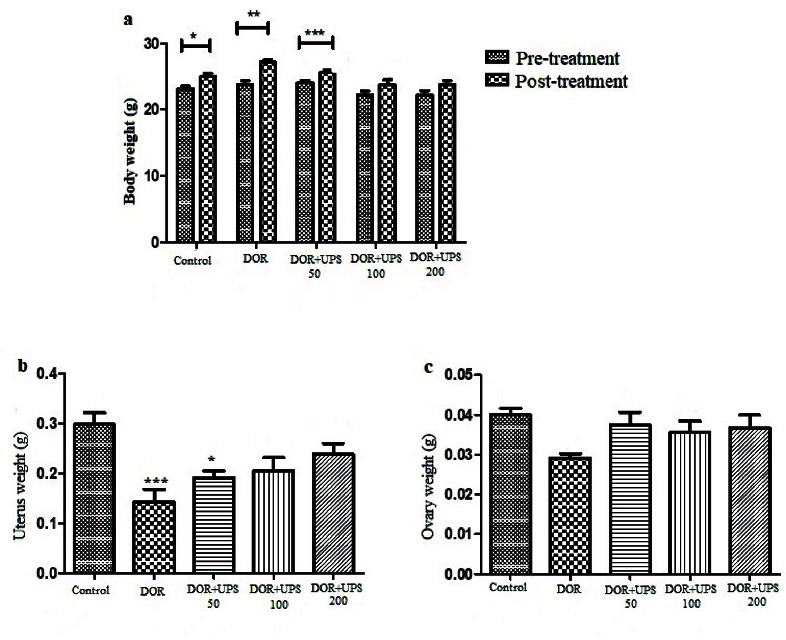
The analysis of A) Body, B) Uterus, and C) Ovary weight of diminished ovarian reserve (DOR) mice after *U. pilulifera* seed extract (50, 100, 200 mg/kg) administration for 14 days. *P < 0.05, **P < 0.01, ***P < 0.001, vs. control group.

The body, uterus, and ovarian weight were recorded continuously. As indicated in figure 1A, the body weight in the DOR group increased significantly after 14 days compared to the initial weight (p < 0.001). At the same time, the body weight was not affected after UPS administration (p = 0.7). The uterus weight reduced significantly in the DOR group, as seen in figure 1B, compared to the control group (p < 0.001). However, the ovary weight was not affected in different groups (p = 0.08) (Figure 1C).

### The UPS effects on FSH, LH, and E2

**Figure 2:**
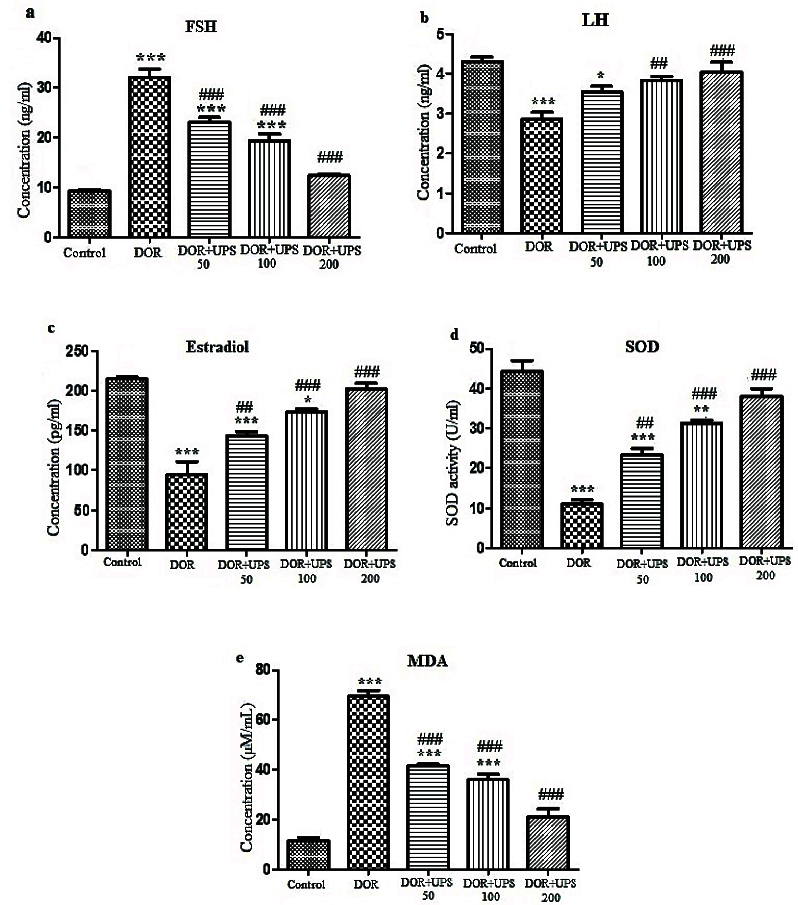
The serum levels of A) FSH, B) LH, C) E2, D) SOD activity, and E) MDA concentration in DOR model mice after UPS administration for 14 days. FSH: Follicle-stimulating hormone, LH: Luteinizing hormone, E2: Estradiol, SOD: Superoxide dismutase and MDA, malondialdehyde, DOR: Diminished ovarian reserve, UPS: *U. pilulifera* seed extract (50, 100, 200 mg/kg). *P < 0.05, **P < 0.01, ***P < 0.001 vs. control group, ${\;}^{\#\#}$ P < 0.001, ${\;}^{\#\#\#}$ P < 0.001 vs. DOR model group.

The serum levels of FSH, LH, and E2 changed significantly in the DOR groups (Figure 2). The levels of FSH (Figure 2A) increased significantly, while LH (Figure 2B) and E2 (Figure 2C) levels showed a significant reduction in the DOR group compared to the control group (p < 0.001).

However, after 14 days of treatment with UPS, the serum levels of FSH were reduced in a dose-dependent manner compared to the DOR group (p < 0.001). In addition, the serum LH and E2 levels increased in the UPS groups compared to the DOR group in a dose-dependent manner (LH; 100, 200 mg/kg, p = 0.01 and E2; 50, 100, 200 mg/kg, p = 0.01, p < 0.001, p < 0.001, respectively).

### The effects of UPS on oxidative stress markers

To determine the effect of UPS administration on oxidative stress, the SOD activity and MDA levels were assessed in the serum of Balb/c mice. As illustrated in figure 2, the SOD activity decreased (Figure 2D) and the MDA concentration (Figure 2E) increased significantly in the DOR model compared to the control group (p < 0.001). The administration of UPS for 14 days (all 3 doses) had protective effects on these changes in a dose-dependent manner. UPS increased the SOD activity, especially at doses of 100 and 200 mg/kg (p < 0.001) and reduced the MDA concentration significantly compared to the DOR group (p < 0.001).

### The effects of UPS on ovary and uterus morphometry

**Figure 3:**
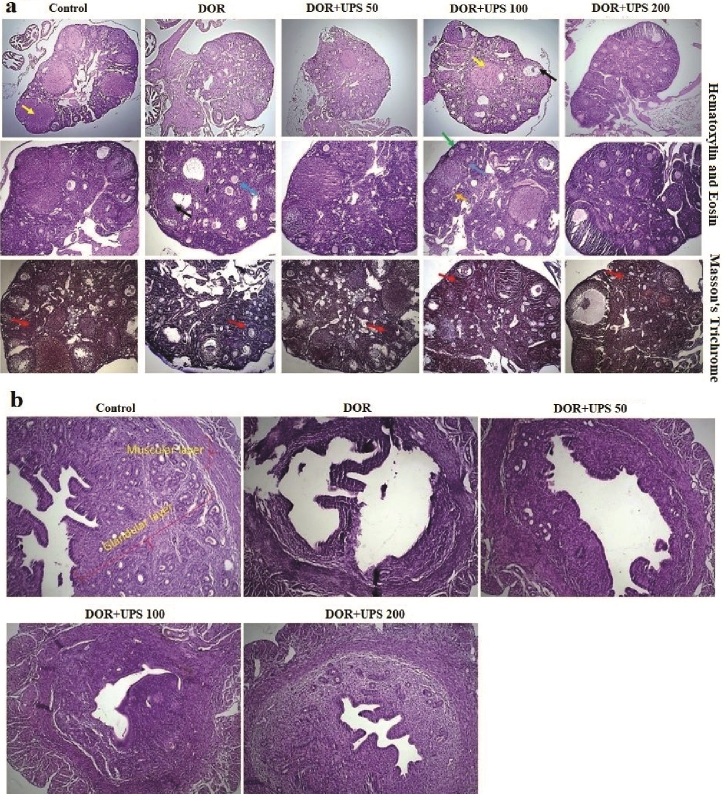
Morphological alterations in ovaries in DOR mice after UPS administration for 14 days. A) H&E and Masson's Trichrome staining of ovaries and B) H&E staining of the uterus in different groups. DOR: Diminished ovarian reserve, UPS: *U. pilulifera* seed extract (50, 100, 200 mg/kg). Red: Atretic follicles, black: Graffs, yellow: Corpus luteal, blue: Secondary follicle, green: Primordial follicle, and orange arrow: Primary follicle, scale bar = 200 μm.

**Figure 4:**
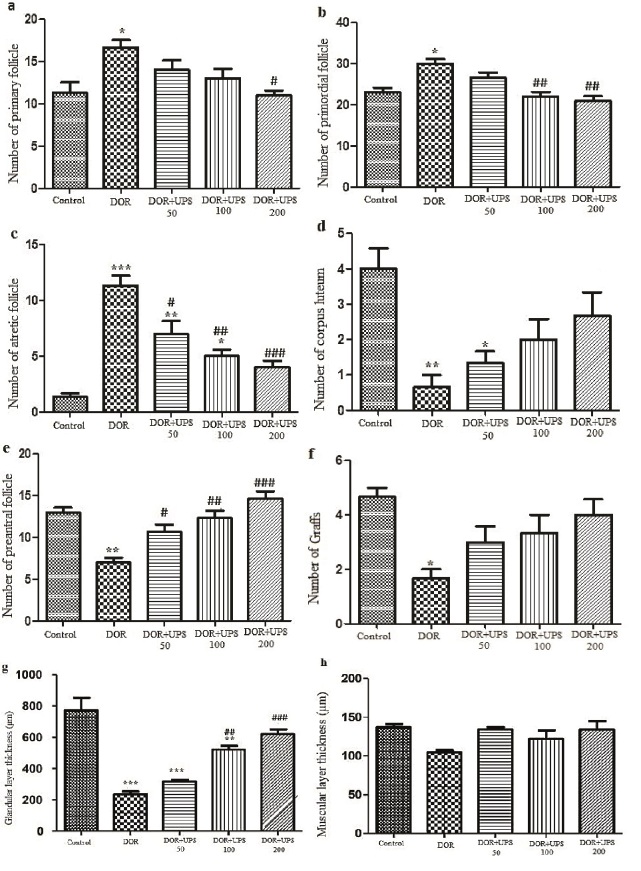
The number of the A) Primary, B) Primordial, C) Atretic follicles, D) The corpus luteum, E) Preantral follicles, F) Graffs in ovary tissues, G) Thickness layer of glandular, and H) Muscular in uterus tissues in DOR model balb/c mice and after UPS treatment for 14 days. DOR: Diminished ovarian reserve, UPS: *U. pilulifera* seed extract (50, 100, 200 mg/kg). *P < 0.05, **P < 0.01, ***P < 0.001 vs. control group, ${\;}^{\#}$ P < 0.05, ${\;}^{\#\#}$ P < 0.01, ${\;}^{\#\#\#}$ P < 0.001 vs. DOR model group.

To evaluate the impact of UPS on the morphometry of the ovaries and uterus, histological analysis (Figures 3A, B) was conducted to assess various features. These included the quantification of primordial, primary, preantral, and atretic follicles, as well as the number of Graffs and corpus luteum. Additionally, the thickness of the muscular and glandular layers of the uterus was measured. As shown in figure 4 A-C, the number of primordial, primary, and atretic follicles increased significantly in the DOR model groups compared to the control group (p = 0.01, p = 0.01, and p < 0.001, respectively). On the contrary, the number of corpus luteum, preantral follicles, and Graff showed a significant reduction in the model group when compared to the control group (Figure 4D-F, p = 0.01, p < 0.01, and p = 0.02, respectively).

The administration of UPS at a dose of 200 mg/kg improved the number of primary follicles compared to the DOR group (p = 0.02). The number of primordial and atretic follicles was significantly reduced by UPS in a dose-dependent mode in comparison to the DOR group (Figure 4B, C).

UPS, when administered at doses of 50, 100, and 200 mg/kg, significantly increased the number of preantral follicles in a dose-dependent manner compared to the DOR group (Figure 4E, p = 0.04, p < 0.01, and p < 0.001, respectively).

The thickness of the muscular and glandular layers of uterus tissues was measured. As seen in figure 4G, the thickness of the glandular layer decreased significantly in the DOR group compared to the control group. Administration of UPS increased the thickness of the glandular layer compared to the DOR group in a dose-dependent manner (100, 200 mg/kg; p = 0.01 and p < 0.001, respectively). However, no changes were observed in the thickness of the muscular layer in different groups (p = 0.09) (Figure 4H).

### UPS effects on ovarian cell apoptosis

**Figure 5:**
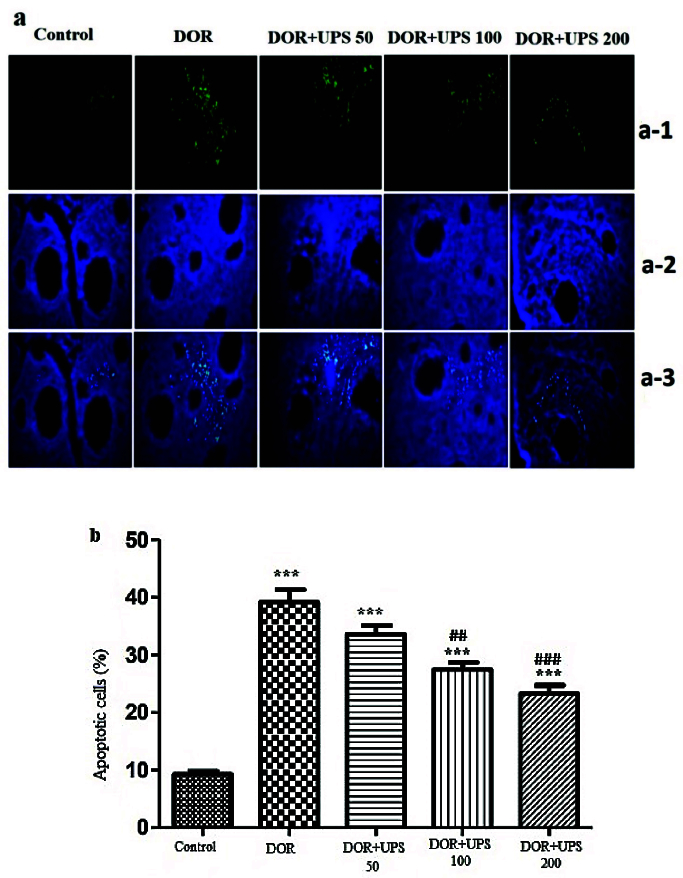
Anti-apoptotic effect of UPS treatment on the cyclophosphamide-induced DOR in mice. A) Analysis of apoptosis by TUNEL staining in ovaries. Counterstaining was performed using DAPI nuclear staining and pictures were taken using A-1) FITC, A-2) DAPI, and A-3) Merged filters. Scale bar = 200 μm. B) The percentage of apoptotic cells in various groups. DOR: Diminished ovarian reserve, UPS: *U. pilulifera* seed extract (50, 100, 200 mg/kg). ***P < 0.001 vs. control group, ${\;}^{\#\#}$ P < 0.01, ${\;}^{\#\#\#}$ P < 0.001 vs. DOR model group.

To analyze the apoptotic cell alterations in the DOR model and assess the potential protective effects of UPS, the ovaries were removed from the mice and utilized for the TUNEL assay. As shown in figure 5A, the proportion of apoptotic cells increased significantly in the DOR group (p < 0.001) compared to the control group. However, this ratio decreased markedly after the administration of UPS in a dose-dependent manner (100, 200 mg/kg, p = 0.01 and p < 0.001, respectively) (Figure 5B).

**Table 1:** Fatty acids and sterol composition of *U pilulifera* seed oil by GC-MS


**Number**	**RT (min)**	**Area%**	**Name**
**1**	13.852	0.30	Thymol
**2**	27.031	10.36	n-Hexadecanoic acid
**3**	28.795	0.08	9,12-Octadecadienoic acid (Z, Z)-, methyl ester
**4**	29.973	59.25	Linoleic acid
**5**	30.051	8.29	Oleic acid
**6**	30.248	6.06	Vanicol
**7**	31.659	0.16	15-Hydroxypentadecanoic acid
**8**	33.262	0.22	Linolein
**9**	33.366	0.06	9,12-Octadecadienoic acid (Z, Z)-
**10**	33.47	0.09	n-Tetracosane
**11**	33.911	0.61	Bicyclo [10.1.0]tridec-1-ene
**12**	33.963	0.19	9,12-Octadecadienoic acid (Z, Z)-
**13**	34.01	0.29	Olealdehyde
**14**	34.362	0.40	Anthraquinone
**15**	34.767	0.09	Undecane

**Number**	**RT (min)**	**Area%**	**Name**
**16**	34.84	0.12	VestinolAH
**17**	35.12	0.06	9,17-Octadecadienal, (Z)-
**18**	36.018	0.08	Eicosane (CAS) n-Eicosane
**19**	36.594	6.47	Linolein
**20**	36.645	2.61	Glyceryl Monooleate
**21**	37.45	0.10	Benzothieno
**22**	38.762	0.12	Propane, 1,3-dicyclohexyl-2-methyl
**23**	39.499	0.21	Tetracosane
**24**	40.807	0.56	Gamma-Tocopherol
**25**	41.777	0.18	Eicosane
**26**	43.116	0.23	Campesterol
**27**	44.579	1.52	22,23-dihydro-Stigmasterol
**28**	44.771	0.34	20-methylene-pregn-5-en-3beta-ol
**29**	45.051	0.22	alpha-Amyrin
**30**	45.58	0.18	1,4-Dihydro-9-isopropylidene-5,6,7,8-tetramethoxy-1,4-methanonaphthalene
**31**	45.809	0.53	Viminalol
RT: Retention time (min)			

## 4. Discussion

The assessment of ovarian reserve plays a crucial role in predicting female fertility. In addition to conventional medications, utilizing natural-origin products, such as herbal extracts, is particularly appealing due to their perceived safety and affordability. This study aimed to investigate the effects of different doses of UPS in a CTX-induced DOR model.

The results revealed a decrease in the weight of the ovary and uterus in the DOR model. However, UPS administration led to an increase in uterus weight in a dose-dependent manner. Considering that sex hormones, such as FSH, LH, and estradiol, play an important role in regulating fertility, their levels were measured in various study groups to assess the potential effects of UPS.

The results demonstrated a significant increase in FSH levels in the model group, accompanied by a decrease in LH and E2 concentrations compared to the control group. Notably, our findings indicated that UPS administration reduced FSH and increased LH and E2 levels in a dose-dependent manner. A study conducted by Huang et al., supports our findings, as they observed that the administration of CTX at a single dose of 90 mg/kg resulted in a significant decrease in E2 levels and an increase in FSH levels (6). These results align with our findings, indicating that UPS has the potential to modulate the altered hormone profile caused by CTX administration.

DOR is a multifactorial disorder influenced by various conditions, such as genetic factors, oxidative stress, and DNA damage. Oxidative stress disturbs fertility through multiple pathways, including the induction of DNA and mtDNA damage. Previous studies have underscored the impact of ROS on female fertility and DOR (24–26). In the DOR model, the activity of SOD, an antioxidant enzyme responsible for scavenging superoxide radicals, was observed to decrease. However, our findings indicated that the administration of UPS increased the SOD activity in a dose-dependent manner. Additionally, malondialdehyde (MDA), a marker of lipid peroxidation, exhibited elevated levels in the DOR model group (27). In contrast, our results demonstrated that UPS administration mitigated MDA concentration compared to the DOR group in a dose-dependent manner.

The antioxidant effects of *U. pilulifera* leaf extract have been demonstrated in a previous study by Amawi et al., on pre-diabetic Wistar rats. The results indicated that the extract significantly reduced MDA levels while increasing the levels of glutathione (GSH) and total antioxidant capacity (TAC) (28).

Our findings revealed the presence of different fatty acids, such as oleic acid, palmitic acid, and linoleic acid, in UPS. These fatty acids have been the subject of various studies, which have consistently demonstrated their beneficial effects on improving fertility (29). Oleic acid, a monounsaturated fatty acid, has been shown to enhance the development of oocytes and embryos and increase the levels of hormones associated with ovulation (30). It is generally recommended for women of childbearing age who are trying to conceive to consume higher amounts of monounsaturated and polyunsaturated fats, including omega-3 fatty acids while limiting their intake of trans and saturated fats (31). Indeed, certain fatty acids, such as linoleic acid, possess anti-inflammatory properties. Previous study reported that these fatty acids can reduce inflammation induced by enzymes like phospholipase A ${\;}_{2}$ (32).

Elevated production of ROS during oxidative stress can lead to DNA damage and apoptosis. Within the follicle, the granulosa cells, including both mural and cumulus cells, are particularly susceptible to apoptosis. These cells play critical roles in the secretion of steroidal hormones and growth factors. Apoptosis in granulosa cells has been linked to diminished quality and quantity of oocytes (33). Fan et al., conducted a study indicating that apoptosis in granulosa cells, particularly in mural cells, is associated with a decline in ovarian reserve. They further noted that the apoptosis rate intensifies in females after the age of 37 (34). Consistent with this, our findings demonstrated CTX-induced apoptosis in the DOR model group. However, the administration of UPS for 14 days exhibited a dose-dependent rescue effect, preventing cell apoptosis in the treated groups.

In addition to granulosa cell apoptosis, abnormal proliferation and differentiation of these cells contribute to the sensitization of follicles to atresia, which is considered an accepted mechanism in the pathogenesis of DOR (35). The histopathological findings from our study revealed a significant increase in the number of primary, primordial, and atretic follicles. Furthermore, the DOR model groups exhibited a significant reduction in the number of corpus luteum, preantral follicles, and Graffs when compared to the control group. The administration of UPS demonstrated its potential to alleviate the reduction in the number of primary, atretic, and primordial follicles compared to the DOR group. Additionally, UPS administration increased the number of preantral follicles in a dose-dependent manner.

CTX, as an alkylating agent, exerts its effects on ovarian follicles through various pathways, including DNA damage-induced apoptosis, and the production of ROS. It functions as a cytotoxic agent, inhibiting cell division and leading to the reduction of ovarian follicles. The findings from previous studies suggest that the loss of ovarian reserve is associated with the enhanced activation of primordial follicles. This increased activation of primordial follicles ultimately leads to the depletion of the follicle pool, contributing to the decline in ovarian reserve (36, 37). Currently, there is no effective or known method to treat DOR, so this study did not use a positive control; and this was the limitation of our study.

## 5. Conclusion

The present study demonstrated that the administration of UPS had a beneficial impact on the loss of ovarian follicles. The findings revealed that UPS exhibited the ability to alleviate oxidative stress and apoptosis in the ovaries, while also regulating the levels of steroidal hormones such as FSH, LH, and E2 in a dose-dependent manner. Furthermore, the results obtained from the histopathological analysis provided evidence supporting the therapeutic potential of UPS extract in the DOR model induced by CTX. Based on the phytochemical analysis, it can be inferred that the beneficial effects of UPS may be attributed to the presence of specific fatty acids, including oleic acid, palmitic acid, and linoleic acid. However, it should be noted that this conclusion cannot be extrapolated to other etiological causes of DOR. The results of the present study introduce a new potential treatment option for patients.

##  Data availability

Data supporting the findings of this study are available upon reasonable request from the corresponding author.

##  Author contributions 

SH and SNSL had full access to complete data in the study and took responsibility for the integrity of the data and the accuracy of the data analysis. Concept and design: MSh, SNSL, TT, MMA, MT. Acquisition, analysis, or interpretation of data: SH, RSK, and MMA. Drafting of the manuscript: SH, RSK, and MT. Critical revision of the manuscript for important intellectual content: all authors. Supervision: SNSL, MSh, TT.

##  Acknowledgments

This study has been funded and supported by Tehran University of Medical Sciences, Tehran, Iran (Grant no. 48936–147-2–99).

##  Conflict of Interest

The authors declare that there are no conflict of interest.
